# Sequoia: an interactive visual analytics platform for interpretation and feature extraction from nanopore sequencing datasets

**DOI:** 10.1186/s12864-021-07791-z

**Published:** 2021-07-07

**Authors:** Ratanond Koonchanok, Swapna Vidhur Daulatabad, Quoseena Mir, Khairi Reda, Sarath Chandra Janga

**Affiliations:** 1 Department of Human-Centered Computing, School of Informatics and Computing, Indiana University Purdue University, 535 W Michigan St, Indianapolis, IN 46202 USA; 2 Department of BioHealth Informatics, School of Informatics and Computing, Indiana University Purdue University, 719 Indiana Ave Ste 319, Walker Plaza Building, Indianapolis, Indiana 46202 USA; 3 Department of Medical and Molecular Genetics, Medical Research and Library Building, Indiana University School of Medicine, 975 West Walnut Street, Indianapolis, Indiana 46202 USA; 4 Centre for Computational Biology and Bioinformatics, 5021 Health Information and Translational Sciences (HITS), Indiana University School of Medicine, 410 West 10th Street, Indianapolis, Indiana 46202 USA

**Keywords:** RNA modifications, Epitranscriptome, Single-Molecule Sequencing, Nanopore signal analysis, Visual infrastructure, Next-generation-sequencing, Bioinformatics

## Abstract

**Background:**

Direct-sequencing technologies, such as Oxford Nanopore’s, are delivering long RNA reads with great efficacy and convenience. These technologies afford an ability to detect post-transcriptional modifications at a single-molecule resolution, promising new insights into the functional roles of RNA. However, realizing this potential requires new tools to analyze and explore this type of data.

**Result:**

Here, we present Sequoia, a visual analytics tool that allows users to interactively explore nanopore sequences. Sequoia combines a Python-based backend with a multi-view visualization interface, enabling users to import raw nanopore sequencing data in a Fast5 format, cluster sequences based on electric-current similarities, and drill-down onto signals to identify properties of interest. We demonstrate the application of Sequoia by generating and analyzing ~ 500k reads from direct RNA sequencing data of human HeLa cell line. We focus on comparing signal features from m6A and m5C RNA modifications as the first step towards building automated classifiers. We show how, through iterative visual exploration and tuning of dimensionality reduction parameters, we can separate modified RNA sequences from their unmodified counterparts. We also document new, qualitative signal signatures that characterize these modifications from otherwise normal RNA bases, which we were able to discover from the visualization.

**Conclusions:**

Sequoia’s interactive features complement existing computational approaches in nanopore-based RNA workflows. The insights gleaned through visual analysis should help users in developing rationales, hypotheses, and insights into the dynamic nature of RNA. Sequoia is available at https://github.com/dnonatar/Sequoia.

**Supplementary Information:**

The online version contains supplementary material available at 10.1186/s12864-021-07791-z.

## Background

In recent years, several studies have led to the discovery of dynamic chemical modifications of nucleotide RNA bases, which are changes that are increasingly seen as key switches in the metabolism of RNA [1–3]. While modifications like pseudo uridine (ψ) and internal N6-methyladenosine (m6A) in mRNAs have been known for decades [4], lack of efficient detection and analysis techniques limited their transcriptome-wide profiling, impeding the field of epitranscriptomics.

Single-molecule, long-read sequencing technologies from Oxford Nanopore Technologies (ONT), however, is enabling cost-effective RNA sequencing. The MinION from the ONT, for example, records changes in the current across the nanopore as an RNA molecule passes through a nanopore. The current disruption is sensitive to the characteristic of the moiety passing through the pore, making it possible to detect modifications at single-base level. However, developing tools that can automatically detect such modifications is a challenge, since it requires an understanding of signal features that discriminate modified from non-modified bases. Although such analyses can greatly benefit from visual inspection of signal features currently there are limited tools and resources which can facilitate the visual exploration of the signal space, impeding the development of computational models for single molecule mapping of modifications.

Recently multiple packages and frameworks became available for analyzing and visualizing nanopore sequencing data (e.g. poRe [5], poretools [6], HPG Pore [7], NanoPack [8], NanoPipe [9], and NanoR [10]), however most of these existing tools are primarily designed to provide summary visualizations or descriptive statistics of an overall run. These tools provide limited support for analyzing signal features at a detailed level. There is thus an unmet need for new visualization techniques to enable detailed visual exploration of ONT signal characteristics while supporting the comparison of large multiples of signals. Even though tools that enable users to explore signal information do exist, such as SquiggleKit [11], which enables users to compare raw signal traces with a given nucleotide sequence using the dynamic time warping algorithm, they do not allow users to look into the individual signal instance of each read, nor render options to observe signal clusters based on signal similarity.

In this study, we present Sequoia, a visualization tool for exploring signals generated by the ONT. Sequoia was specifically designed to aids users in interactively exploring nanopore current signals underlying RNA sequences with the aim of finding features that differentially discriminate modified from unmodified bases. The tool enables users to directly import Fast5 files generated by the ONT and employs dynamic time warping to cluster signals based on the similarity of their electric current or the sequences they represent as identified by base-calling algorithms. Sequoia provides a multi-view interface for nanopore signal analysis at both an overview and very detailed level. The software consists of a command-line Python-based component for data processing and a visualization that can be run within a web browser. We deploy the Sequoia pipeline to compare nanopore RNA signal data of modified and unmodified bases. We show how interactive visualization enables the discovery of qualitative signal that discriminate m6A and m5C modifications from otherwise nonmodified RNA bases. Qualitative insights unearthed through Sequoia can suggest features for building automated modification-prediction techniques, paving the way for high-throughput profiling of epitranscriptomic events.

## Methods

**Fig. 1:**
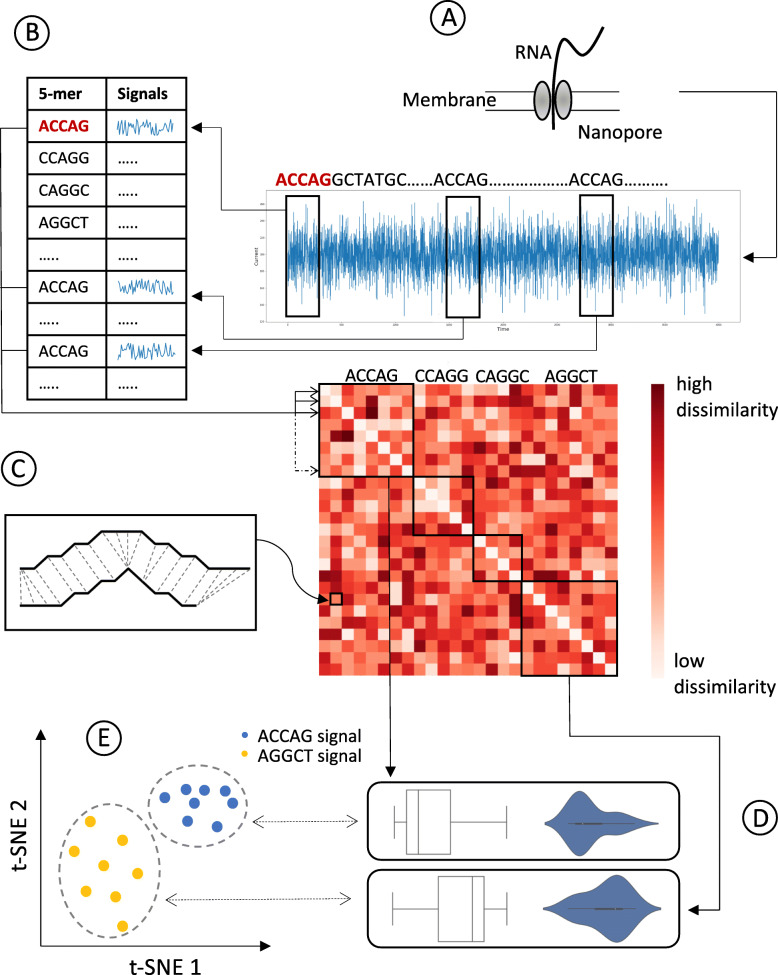
Sequoia’s workflow showing: **A** Nanopore based single molecule sequencing, **B** Extracting signals corresponding to successive 5-mers from input Fast5 file, **C** Constructing a similarity matrix by computing a similarity score for every pair of signal instances using the dynamic time warping algorithm, subset showing DTW based distance between signals being compared, **D** Displaying homogeneity of signal instances within each 5-mer through box and violin plots based on the summary statistics of the similarity matrix (**E**) Illustrating variabilities among signal instances across multiple 5-mers via t-SNE plot

Sequoia is an interactive platform for nanopore sequencing data, which not only facilitates an intuitive visualization but also enables an efficient feature extraction from nanopore sequencing datasets. Sequoia allows comparison of signal instances across various 5-mers and further analyze them in detail to develop a profound understanding of user defined datasets. Initiated via command-line, the long-read data generated from Nanopore goes through a series of backend data processing steps. As seen in Fig. 1A, the user input Fast5 files are parsed into a table of 5-mers and their corresponding signal instance pairs using Sequoia’s signal extraction script implemented in Python (see Backend: computational framework). Furthermore, to compute a similarity matrix that compares every pair of 5-mers (Fig. 1B), a dynamic time warping algorithm is deployed using the Python package called ‘dtaidistance’ (10.5281/zenodo.3981067). Each value in the similarity matrix is the quantification of similitude between any two given signal instances. A lower similarity score indicates more resemblance while a higher score indicates more contrast, thereby leaving all the diagonal values of the similarity matrix as zero since each signal instance is being compared to itself (see Backend: computational framework). Once the preprocessing is completed, Sequoia’s visualization (implemented using Javascript’s library D3.js) can be visualized using a web browser (see GitHub page for detailed instructions). Sequoia’s interface consists of multiple visualizations, each depicting a different level of insight about the input data. Sequoia generates a box/violin plot showing similarity score distributions within each 5-mer, a t-SNE plot (implemented using tsne.js) illustrating similarity between signal instances of up to four user-interested 5-mers, and a line graph displaying the selected signal instances, a snippet of which can be seen in Fig. 1C. The box and violin plots are the representation of how uniform the signal instances for each 5-mer are. If the median and distribution are closer to zero, it implies that the corresponding signals instances of that 5-mer are homogenous. On the other hand, the t-SNE plot on Sequoia can be used to compare signal instances from up to four unique 5-mers. Signal instances with relatively low similarity scores will be clustered together, while signal instances with relatively high similarity score will be distinguishably further. Figure 1D and E show the steps where several statistics are computed, including current averages, medians, and t-SNE projections. These statistics are then used to generate the visualizations.

### Backend: computational framework

Nanopore sequencers generate Fast5 files from sequenced reads, where Fast5 is an hdf5 based hierarchy of directories containing events table, signal information, and other metadata as seen in Supplementary Fig. 1. The events table has successive 5-mers annotated with the indexes of the pertaining values from the signal list. Where, the signal list is an array of electric current values indexed in tandem with the events table (see Supplementary Fig. 1). To process the raw Fast5 data from the Oxford Nanopore sequencer, we developed Python scripts to extract and reformat the signal information for further analysis and visualization.

#### Input file preprocessing and signal extraction

The Sequoia pipeline starts by submitting a Nanopore-generated Fast5 file through a Python-based command-line script. The script parses and extracts the electric current signals for each consecutive 5-mer in the full-length read. This is achieved by extracting the observed electric time-series within a five-nucleotide window. The window is then slid by one nucleotide to the right to obtain the signal for the next 5-mer’s. The process repeats until reaching the end of the read, effectively annotating all overlapping 5-mers in that read. Note because the electric signal for a 5-mer is determined by ‘start’ and ‘stop’ indices in the Nanopore event table, the 5-mers may have different signal length. We refer to each signal (corresponding to a single 5-mer) as a ‘signal instance’. The cumulative list of signals (representing all 5-mers) is hereafter referred to as the ‘cumulative signals’ (see Supplementary Fig. 1). The output cumulative signals are then fed into the rest of the pipeline to compute a similarity matrix, summary statistics, and other 5-mer specific information.

#### Similarity matrix and dynamic time warping

To effectively cluster nanopore signals, we employ dynamic time warping to measure the similarity of 5-mers. We compute a full similarity matrix, measuring the similarity of every pair of 5-mers in the input. Each numerical value in the matrix is a representation of how similar two corresponding signals are. The smaller the value, the more similarity there is between the two signals. Dynamic time warping enables measuring similarities between two temporal sequences that may vary in length [12]. If A and B are two nanopore current timeseries representing two 5-mers, Dynamic Time Warping (DTW) is defined as:

1 $$\mathrm A={\mathrm a}_1,\;{\mathrm a}_2,\;{\mathrm a}_3,\dots..,\;{\mathrm a}_{\mathrm n}$$

2 $$\mathrm B={\mathrm b}_1,\;{\mathrm b}_2,\;{\mathrm b}_3,\dots..,{\mathrm b}_{\mathrm m}$$

3 $$Cost\left(w\right)= \sum _{k=1}^{K}d\left({w}_{k}\right)$$

4 $$DTW\left(A,B\right)=\text{m}\text{i}\text{n}\left(Cost\left(w\right)\right)$$

Where w_k_ is the matrix element belonging to the k^th^ element of the warping path W that represents a mapping between A and B [12]. In other words, dynamic time warping works by warping sequences A and B to A* and B* such that the dissimilarity (i.e. cost) between A* and B* is minimized. “Warping” includes edits, such as expanding or contracting the timeseries, in order to find the best alignment between the two signals. If the penalty is not zero, each edit will be considered a cost. Specifically, the penalty term will be added to Eq. (3) as follows.

5 $$Cost\left(w\right)= \sum _{k=1}^{K}d\left({w}_{k}\right)+p \sum _{k=1}^{K}I$$

Where p is the specifiable penalty that will be added each time an expansion or contraction occurs (i.e. when I = 1, otherwise I = 0).

Our use of DTW is motivated by the fact that it offers more tolerance in comparing signals, compared to a straight matching criterion. Additionally, DTW allows for measuring similarity between signals of different lengths, which is necessary in our case, given that 5-mers could have signals that vary in length, which is determined by the time needed for the molecule to pass through the nanopore. The constructed similarity matrix comprises the raw signal similarity between every pair of 5-mers found in the run. The matrix serves as an input to the clustering algorithm and visualization.

### Frontend: visualization

#### Interface components

**Fig. 2:**
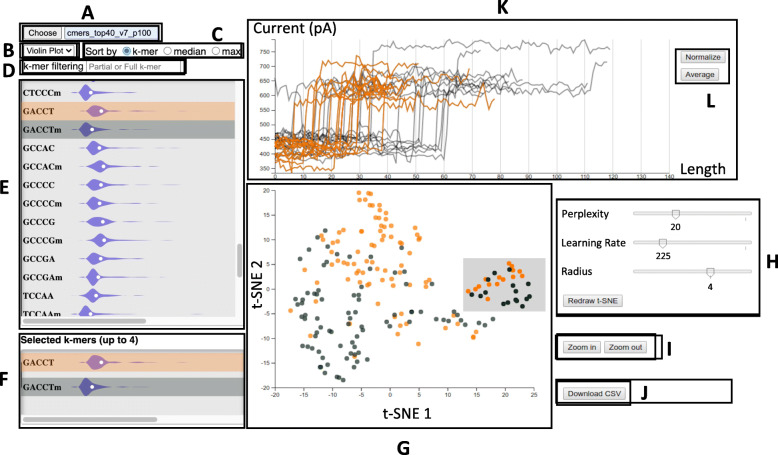
Sequoia’s interface overlay: **A** Textbox for specifying input data directory, **B** Dropdown menu for selecting either a box or violin plot, **C** Available sorting options for the box/violin plots, **D** Textbox for filtering box/violin plots based on 5-mer sequence, **E** Box/violin plots for all 5-mers in the dataset, **F** Box/violin plots for the selected 5-mers, **G** t-SNE plot corresponding to the selected 5-mer, **H** Parameters for adjusting the t-SNE plot, **I** Buttons for zooming in and out of the t-SNE plot, **J** Button for downloading selected t-SNE points, **K** Signal plot corresponding to the selected points on the t-SNE plot, **L** Buttons for normalizing and averaging signal instances

The Sequoia interface consists of a multi-view visualization with several, interactive visual representations. The initial interface consists of a data selection textbox used to upload a preprocessed file and the 5-mer list displaying all 5 nucleotide sequences contained in the file. The charts include a t-SNE plot that visually displays the resemblance of the signal instances in selected 5-mers, a box or violin plot which depict the uniformity across the signal instance of a 5-mer, and a raw signal graph which superimposes signal instances from various 5-mers for comparison. Figure 2 illustrates the various components; each will be described in detail.

i) Data Selection Widget:

The user initiates the visualization by providing the input directory containing the preprocessed Nanopore files, and then clicking the ‘choose’ button, as shown in Fig. 2A. If no directory is provided, Sequoia by default looks for a directory named ‘data’. Multiple directories can be specified by typing them one by one in a textbox and click ‘choose’ after each, enabling comparison between different datasets, or between different dynamic time warping parameter settings.

ii) 5-mer List:

To illustrate the uniformity of signals within 5-mers, Sequoia plots distributions of time warping distances (i.e., signal dissimilarity) in the form of box or violin plots, and ranks those plots in a scrollable list, as seen in Fig. 2E. The distribution of distances is shown by 5-mer, giving the user an overview of signal consistency within each 5-mer class. For instance, in the box plot view in Fig. 2E, the user observes that the distribution of distances for 5-mer GCCAC is generally narrower than 5-mer CTCCC, implying that the latter exhibits more variation in the shape of its signals. The user can interactively switch between box and violin plot via a drop-down menu shown in Fig. 2B. The 5-mers can also be ranked alphabetically, or based on the median distance amongst the signal instances (5-mer with higher signal uniformity appear higher in the list), as illustrated in Fig. 2C. Moreover, the list of 5-mers can be filtered by typing the label of a 5-mer of interest (Fig. 2D), which restricts the list to visualize the distribution of the 5-mers matching the label. Sequoia provides an autocomplete for the search field. Additionally, wild card regular expression can also be used in the search box. For instance, an asterisk act as a wildcard for all four RNA nucleotides (A, C, G, and T); typing ‘AACA*’ brings up AACAA, AACAC, AACAG, and AACAT.

iii) t-SNE plot:

While the distribution plots in the 5-mer list serve as an overview, the t-SNE plot allows a user to study each 5-mer in more detail. The plot visualizes signal similarity on a 2D scatterplot, enabling the comparison of signals from one or more 5-mers. Once a 5-mer is selected from the list, points representing signals will appear in the t-SNE plot. The positioning of the points is such that similar signals appear relatively close to one another, as compared to signals that exhibit a different electric pattern, which are positioned further apart (see Fig. 2G). The positioning of points is determined by the t-SNE algorithm, which is a technique for visualizing high-dimensional data by mapping data points onto a two-dimensional map [13], and based on the similarity matrix using a DTW measure (See Similarity matrix and dynamic time warping for more detail). As illustrated in, a set of t-SNE parameters are available including the adjustable slide bars for the t-SNE’s perplexity, t-SNE’s learning rate, and the circle size to be displayed on the plot. Adjusting these parameters affects how the t-SNE plot will display. For closer inspection of data points, the user can zoom in onto the t-SNE scatterplot (see Fig. 2I). Individual signals can be selected and exported from this plot into a CSV file (Fig. 2J), which provides a way for the user to obtain direct current values for signals of interest.

iv) Signal plot:

The signal plots (Fig. 2K) visualize the raw signal as a line graph representing the raw Nanopore- generated electric current. The X-axis represents time (for the passage of through the Nanopore), whereas the Y-axis represents the strength of the current measured. Multiple signals of the user’s choice (see Interactions across views for more detail) can be superimposed in the signal plot, enabling more detailed examination of signal features. There are two dynamic options for this plot that users can choose in between: normalized and average (Fig. 2L). By default, each signal instance will be displayed with its original length. A normalization option homogenizes the length of the signals in the time dimension, enabling comparison regardless of the time it took for the sequence to pass across the nanopore. The ‘average’ mode groups signals corresponding to the same 5-mer and displays their average, rather than by individual signal. In addition to plotting the median signal, a shaded shows the 25th and 75th percentile of electric current values recorded at each time step.

#### Interactions across views

Up to four 5-mers can be selected simultaneously from the 5-mer list to visualize associated data simultaneously. Once a 5-mer is selected by clicking, its set of signals are added to the t-SNE plot. The colors of points in the scatterplot are consistent with the selection highlight appearing in the box/violin plot. Brushing and linking are also enabled between the views; selecting items from one view will cause the other views to highlight as well. For example, hovering over 5-mers in the list will also cause their signals to be highlighted in the t-SNE and signal plots, thereby revealing the clustering of signals within 5-mer, as well as their general shape. Similarly, hovering over a point in the t-SNE plot will highlight its corresponding signal in the signal plot. Additionally, the t-SNE plot also supports multiple selections; the user can create a ‘brush’ by drawing a rectangular selection onto the plot, causing signals within the bounding box to be selected and highlighted.

### Case studies

To demonstrate the application of Sequoia, we deployed it over two case studies. Each of the case studies illustrated the differential nature of the signal from ONT across m5C and m6A RNA modifications.

#### Motivation

More than 160 types of RNA modifications have been reported, with amalgamating evidence for their role in gene regulation, cell development, translation, metabolism and stress response [14, 15]. However, m6A modification is thought to be the most abundant of mRNA modifications, accounting for the modification of 0.1–0.5 % of all adenosines, with a crucial role in regulating RNA stability, expression, and localization [1, 16, 17]. Although the precise location of m6A modifications on mRNA is still under debate, new high-throughput detection techniques point to their enrichment near 3’ untranslated regions (UTRs) and at stop codons in long exons [18–20]. N5-methylcytidine (m5C) modifications of regular cytosines on RNA were originally observed in tRNAs and rRNAs. It is now known to play a key role in controlling the secondary structure conformation and translation of RNAs [21]. RNA modifications affect diverse eukaryotic biological processes, and the correct deposition of many of these modifications is required for normal development [22]. Therefore, the effective detection of these modifications from direct sequencing data could provide a wealth of useful data for understanding functional RNA roles.

We used Sequoia to explore modified vs. unmodified samples in order to understand differential signals from ONT-based long-read sequencing. Corresponding visualizations were generated to establish the application, scalability and scope of the tool.

#### Data generation

##### Cell culture

Hela cell line was purchased from ATCC cell line collection and cultured in DMEM media supplemented with 10 % FBS and 0.5 % penicillin/streptomycin.

##### Library preparation and RNA sequencing

Libraries were prepared following the Nanopore Direct RNA sequencing kit documented protocol (SQK-RNA002). Briefly, total RNA was isolated using Qiagen RNeasy Mini Kit (Cat No. /ID: 74,104), followed by PolyA enrichment using Thermo Fisher Dynabeads™ mRNA DIRECT™ Micro Purification Kit (61,021). 500 ng of poly (A) RNA was ligated to a poly (T) adaptor using T4 DNA ligase. Following adaptor ligation, the products were purified using Mag-Bind® TotalPure NGS Beads (M1378-00), following the NGS bead purification protocol. Sequencing adaptors preloaded with motor protein were then ligated onto the overhang of the previous adaptor using T4 DNA ligase followed by NGS bead purification protocol. The RNA library was eluted from the beads in 21 µl of elution buffer and quantified using a Qubit fluorometer using the manufacturer’s RNA assay. The final RNA libraries were loaded to FLO-MIN106 flow cells and run on MinION. Sequencing runs and base calling were performed using MinKNOW software (Oxford Nanopore Technologies Ltd.) The data output from MinKNOW for Hela cell line was 800k sequence reads using one flow cell. MinKnow generates data as pass and fail folders. FAST5 files from pass folder were again base called using Albacore 2.1.0 (Oxford Nanopore Technologies Ltd.) resulting in 500 K single-molecule reads for the Hela cell line which corresponded to full-length transcripts ranging from 50b to 8 kb. The direct RNA-sequencing data generated in this study are publicly available on SRA, under the project accession PRJNA604314.

#### Data processing

##### Modification location-specific signal extraction

To visually compare the ONT generated signal across modified and unmodified RNA bases, we extracted 8737 genomic locations for m5C and 84,149 m6A modifications in HeLa cells from previous RNA modification studies [23–26]. Adhoc scripts available on the github repository of Sequioa were used to complete the location-based signal extraction process. Briefly, these scripts encapsulate the following steps. The first step of the signal extraction process was to index the organization of the Fast5 files for the HeLa dataset generated in this study. This indexing information was fed into the downstream to efficiently locate and navigate the Fast5 files. Simultaneously, we used Guppy (V 4.4.1) (https://nanoporetech.com) to base call the Fast5 data generated and aligned the corresponding Fastq to the hg38 human genome using Graphmap (V 0.5.2) [27]. The resulting sam files were then processed along with the RNA modification genomic coordinates to extract the read index coordinates. All this information was then fed into the in-house developed signal extraction pipeline (see Input file preprocessing and signal extraction) to extract the signal corresponding to the 5-mer surrounding each of the experimentally known m5C and m6A modified locations. Similarly, signals corresponding to 5-mers for an equal number of random unmodified genomic locations where the middle base is a C or an A nucleotide were also extracted for unbiased comparison.

## Results

Long-read direct RNA sequencing promises a potential to answer fundamental questions around cellular components’ interaction and function from a transcriptomic and clinical point of view [28, 29]. Nanopore sequencing technology has shown particularly promising progress on this front. As more and more research groups adopt long-read sequencing, there is increasing need to understand and develop insights about the data generated by these technologies. Despite its rapidly expanding user base, the tools to visualize and dissect the Nanopore-based signal information are few. Nanopore sequencers generate a Fast5 output, which follows a HDF5 based organization. The current Nanopore sequencing protocol writes each sequenced read to a Fast5 file although more recent protocols result in multi Fast5 files. As seen in Supplementary Fig. 1, each Fast5 file contains an indexed signal list, which is an array of electric current values (referred to as signal values), and an events table which records the signal indices pertaining to each 5-mer. Therefore, each 5-mer will have a series of signal values from each read, hereby referred to as signal instance (see Supplementary Fig. 1). Since 5-mers repeat in a read, each 5-mer has a consensus of signal instances, hereby referred to as a 5-mer’s cumulative signal (see Input file preprocessing and signal extraction).

Given the temporal nature of a signal instance, a tool like Sequoia, which can emphasize the underlying insights of a time series data is the need of the hour. Although there are tools that specialize in visualizing time series data, they are not necessarily suitable for nanopore sequencing data exploration. For instance, TimeSearcher is a visualization tool for exploring and forecasting time series data, but does not allow segregation of data into groups [30]. Similarly, TimeFork is another analytics tool for exploring and predicting multivariate time-series data [31], which primarily focuses on the prediction of probable sets of temporal data points rather than providing comprehensive insights about the data itself. Therefore, we develop Sequoia, an automated framework to visualize, compare, and analyze Nanopore-based long-read sequencing signal information. Sequoia enables users to load Nanopore-sequencing datasets, generate plots that render insights into signal information, compare how identical signal patterns from various 5-mers are, and cluster signal instances with similar patterns together. An application like Sequoia will not only aid the development of a hypothesis but will also help design coherent experiments. Briefly, Sequoia is a pipeline developed in Python and JavaScript, which extracts and processes the signal information for various 5-mers from the user-defined input Fast5 files. Furthermore, these extracted signal patterns are compared across various 5-mers using Dynamic Time Warping measure to represent signal similarity. Sequoia then uses the computed statistics and generates a local interface to visualize them in various formats like box plots that depict the uniformity across the signal instances of a 5-mer, line graphs superimposing signal from various 5-mers, and t-SNE plots that enable users to compare and discern signal patterns across 5-mers.

To demonstrate the extent of application and adaptability of Sequoia, we extracted Oxford Nanopore-based direct RNA-sequencing signal information for two RNA modifications, m6A and m5C to compare them with their respective unmodified nucleotide signals. Using these case studies, we also wanted to demonstrate and explore how various parameters dynamically interact and influence the signal visualization.

### Comparison of m5C and unmodified cytosine signals illustrates the significant difference in signal length

To demonstrate the effect of one of Sequoia’s adjustable parameters called dynamic time warping penalty, which is the added distance when compression or expansion is applied to match a pair of signals, we compared the signals extracted from m5C and unmodified C in the HeLa cell line. The two datasets used in this study were from the m5C data consisting of 19,391 5-mer with modified ‘C’ in the middle and the unmodified data consisting of 537,697 5-mer with unmodified ‘C’ in the middle. In this study, we focused only on unique 5-mers that had at least 100 signals in the m5C dataset. The rest of the data was filtered out, with a final residue of 46 unique 5-mers. Further, for each of the 46 5-mers, we randomly sampled 100 signals from each dataset to be compared in the visualization.

**Fig. 3:**
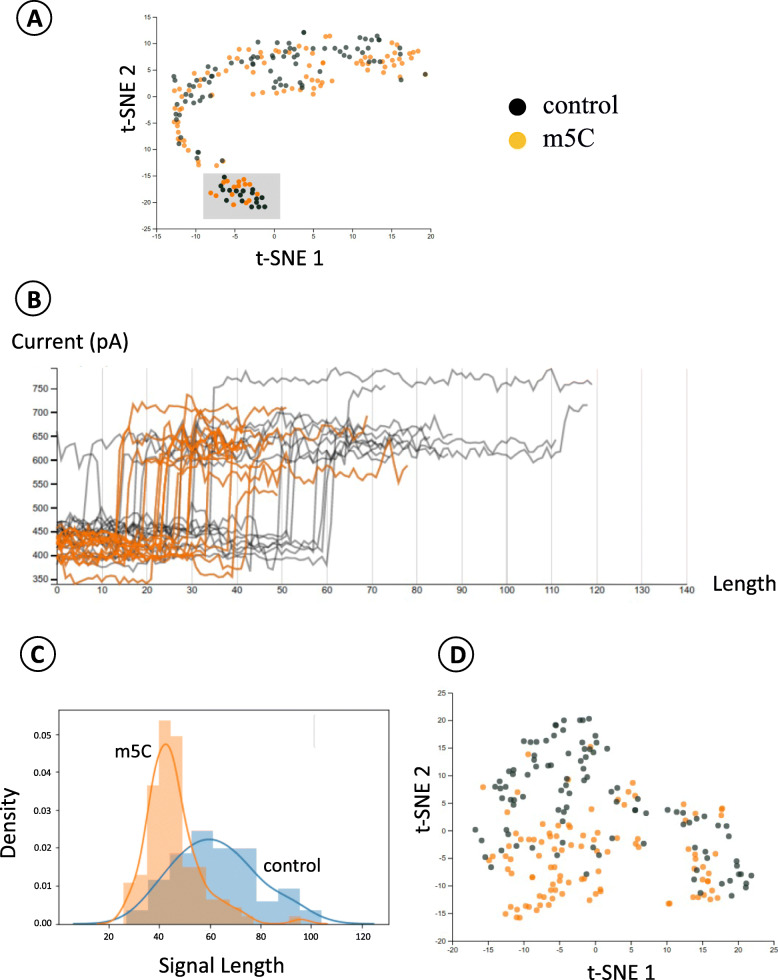
Case study 1: Comparison of the signal instances from modified Cytosine locations (m5C) versus regular Cytosine. **A** Left panel: t-SNE plot of signal clusters for ‘GACCT’ with no dynamic time warping penalty applied. Right panel: Raw signal plot corresponding to the highlighted points in the left panel. **B** Comparing signal length distributions for the regular Cytosine and m5C signals for the 5-mer: GACCT. **C** t-SNE plot for GACCT after applying dynamic time warping with penalty. The contrast across Fig. **3A & 3B** highlight the role of dynamic time warping in segregating and exploring nanopore based signal information

After the preprocessing, we fed the data into Sequoia’s backend and generated the dynamic visualizations shown in Fig. 3. In the first attempt, the dynamic time warping penalty was set to zero. After visualizing the data, we decided to focus on GACCT since it was one of the 5-mers that best showed a promising visual separation between normal and modified signals on the t-SNE plot. It can be seen in the left plot of Fig. 3 A that the points with homogeneous colors are locally grouped. Although the initial result was satisfactory, we sought to further improve the separation between groups.

To explore the data further, we utilized the brushing tool for group selection, which is one of the functionalities provided by Sequoia. By brushing over a group of points on the t-SNE plot (as shown in Fig. 3A), we could see the raw signals associated with the selected 5-mers. We observed that for the GACCT signals that we had sampled, there was a noticeable difference in signal lengths between the normal and modified groups. To confirm that the length difference observed was truly significant, we used the Welch’s t-test to test our sampled data with the null hypothesis that the two populations have equal means. The resulting p-value was less than 0.001, implying that the population means of the two groups were not equal. The distribution plots for both groups are shown in Fig. 3B.

After confirming the significant difference in signal lengths of the two groups, we regenerated the visualization. Given that the signal lengths were largely related to the compression and expansion of signals during the dynamic time warping process, the dynamic time warping penalty was increased to 100, as opposed to 0 in the previous attempt. As a result, the t-SNE plot for GACCT successfully displayed qualitatively better separation between points representing normal and modified signals, as shown in Fig. 3C.

### Large-scale comparison of m6A and unmodified Adenosine using Sequoia demonstrates statistically significant differences in average signal pattern

Sequoia is tailored to answer user-defined questions around how variations in the Nanopore signals can signal functional events or modifications in RNA sequences. For instance, in this case study we demonstrate how differences in the average level of electric current signal can be predictive of m6A modification. We show how these differences can be surfaced by exploring the t-SNE plot and iteratively adjusting the parameters of the visualization.

In order to illustrate Sequoia’s application, we visualized Nanopore-based direct RNA-seq signal for modified (m6A) and unmodified locations in HeLa cell line. After performing the sequencing, signal information for all m6A modified and unmodified locations were extracted (see methods 2.4.2). Since Nanopore generates signal corresponding to a 5-mer passing through the bioengineered pore, rather than single nucleotide, we extracted the signal in such a way that the modified or unmodified Adenosine falls in the middle of the 5-mer. A total of 41,867 5-mers with modified-A in the middle and 1,031,337 5-mers with unmodified-A were extracted. In this study, we focused only on unique 5-mers that had at least 100 signal instances in the m6A dataset. The rest of the data was filtered out, with a final set of 24 unique 5-mers. Furthermore, for each of the 24 5-mers, we randomly sampled 100 signals from modified-A and unmodified-A datasets, to visually compare the signal characteristics using Sequoia.

**Fig. 4:**
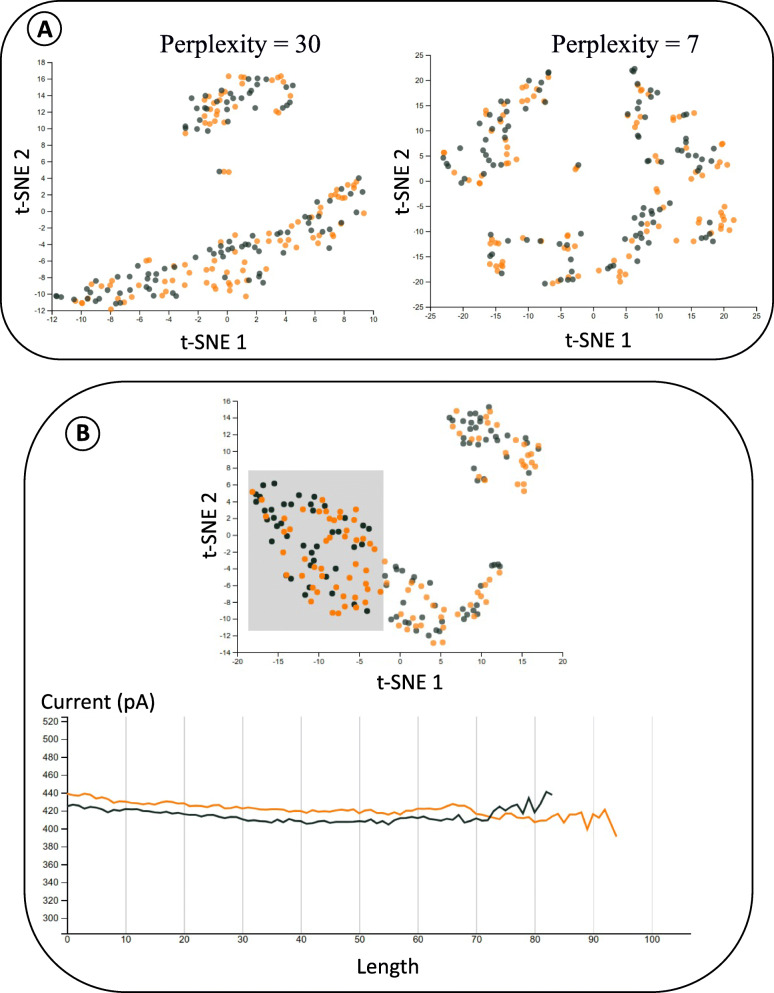
Case study 2: comparing signal patterns based on varying parameters, for modified A (m6A) and a regular Adenosine. **A** Comparing t-SNE plots for ACAGG with perplexity = 30 on the left and perplexity = 7 on the right. Clustering significantly reorders and assembly improves depending on the perplexity and data. **B** On the right panel, the average signal corresponding to the 5-mer ‘ACAGT’ pertaining to the selected points in the t-SNE plot on the left panel. The black line is the average signal of the regular Adenosine reads while the orange is an average signal of the m6A

To visualize the signal disparity across input datasets, Sequoia clusters the signals based on the similarity matrix (see Similarity matrix and dynamic time warping). The cumulative signal instances of above mentioned (modified-A and unmodified-A datasets) were imported into Sequoia and the t-SNE plots are as seen in Fig. 4. When using t-SNE to visualize data, the most important parameter to consider is called perplexity, which is the measure of the effective number of neighbors. A perplexity with a general range of values from 5 to 50 significantly affects the performance of t-SNE. With low perplexity, the points on the t-SNE plot would be scattered. On the other hand, a larger number of points would be grouped when perplexity is high. Figure 4A juxtaposes the t-SNE plots of the signals from ACAGG using high and low perplexity. With higher perplexity, a rough signal pattern can be observed, but only two major groups are distinctively separated. When perplexity is adjusted to be lower, the large groups are broken down into smaller subgroups consisting of similar signals. Higher perplexity is more useful when users would like to consider the big picture of the t-SNE plot, while lower perplexity is more effective if users would like to examine local structures, particularly when there are multiple signal shapes within the same 5-mer. Although dynamic time warping and t-SNE parameters can be adjusted, sometimes observing raw signals from another perspective can lead to more conclusive observations. When inspecting raw signals, Sequoia allows users to change the mode from displaying original signals to displaying the average of a selected group instead. This option can help reveal a difference between the unmodified and modified nucleotide signal group that might be overlooked if not for the averages. For instance, Fig. 4B shows the average signal plot of a group of signals from ACAGT. The t-SNE plot does indicate that there is some separation between normal and modified signals. The signal plot however shows that the modified group has 20 mA higher current value on average for the selected group relative to the unmodified signal group.

## Discussion

Here we demonstrate Sequoia’s ability to distinguish Nanopore sequencing based on signal differences across m6A and unmodified A, and provide the first reports of significant difference in the signal lengths of an m5C and unmodified C in human cell lines. Although applications we demonstrated in are specific to the RNA modifications investigated in this study, Sequoia is not limited to these modifications. Sequoia is developed in such a way that it can be employed to not only study the signal features of raw Nanopore sequencing datasets facilitating the understanding of various types of RNA modifications, such as the Adenosine-to-inosine (A-to-I), Pseudouridine, and 2’-O-methylated nucleotides (Nm), but also potentially to DNA sequencing data, and thereby DNA modifications as well. Sequoia can effectively be used to compare signals of any k-mers of interest across direct RNA, and DNA sequencing-based datasets. Using sequoia users can analyze publicly available Nanopore-based datasets on a large scale, enabling signal-based studies to understand RNA and DNA functional characteristics. Sequoia can also aid in the improvement of the current Nanopore Basecalling accuracy, by comparing various RNA and DNA modifications and discriminating signals to distinguish them from regular bases. Potentially, Sequoia can be developed into a more versatile platform by including a wider range of time-series features and integrating it with machine learning models that can automatically predict modification locations.

## Conclusions

Sequoia is a visual analytics platform that facilitates the exploration of nanopore sequencing datasets. The platform enables users to visualize and cluster signal instances and thereby aid in the identification of RNA modification patterns across datasets. Using Sequoia, users can input the raw Fast5 files of their interest and analyze k-mer specific signals, which can be further processed to generate visualizations that depict the characteristics of datasets as well as k-mers. Sequoia employs algorithms including dynamic time warping and t-SNE to highlight similarities and differences in the underlying nanopore current signal. This framework generates a set of intuitive visualizations, enabling users to pinpoint variations not only across cohorts and datasets but also individual k-mers themselves. We also demonstrate the usability, versatility, and empirical nature of Sequoia by deploying it on two case studies visualizing RNA modifications.

## Availability and requirements

Project name: Sequoia.

Project home page: https://github.com/dnonatar/Sequoia.

Operating system(s): Mac, Linux.

Programming language: Python 3, JavaScript.

Other requirements: web browsers.

License: None.

Any restrictions to use by non-academics: None.

## Supplementary Information

**Additional file 1: Supplementary Figure 1.** Dissection of data units used in the processing of the input FAST5 file. Separated sections showing composition of FAST5 files with Signal list and Events table. Using this Table, 1-mers across each read are annotated with pertaining signals: referred to as Signal instance. All signal instances of 5-mers across reads are condensed into cumulative signals.

## Data Availability

Sequoia is available at the Github repository https://github.com/dnonatar/Sequoia. The visualization demo with pre-loaded m5C data is also available at https://khreda.com/sequoia_demo/index.html. The direct RNA-sequencing data generated in this study is publicly available on SRA, under the project accession PRJNA604314.

## References

[1] Roundtree IA, Evans ME, Pan T, He C (2017). Dynamic RNA Modifications in Gene Expression Regulation. Cell.

[2] Gokmen-Polar Y, Vladislav IT, Neelamraju Y, Janga SC, Badve S (2015). Prognostic impact of HOTAIR expression is restricted to ER-negative breast cancers. Sci Rep.

[3] Neelamraju Y, Hashemikhabir S, Janga SC (2015). The human RBPome: from genes and proteins to human disease. J Proteomics.

[4] Grosjean H (2015). RNA modification: the Golden Period 1995–2015. RNA  (New York, NY).

[5] Watson M, Thomson M, Risse J, Talbot R, Santoyo-Lopez J, Gharbi K (2015). poRe: an R package for the visualization and analysis of nanopore sequencing data. Bioinformatics.

[6] Loman NJ, Quinlan AR (2014). Poretools: a toolkit for analyzing nanopore sequence data. Bioinformatics.

[7] Tarraga J, Gallego A, Arnau V, Medina I, Dopazo J (2016). HPG pore: an efficient and scalable framework for nanopore sequencing data. BMC Bioinform.

[8] De Coster W, D’Hert S, Schultz DT, Cruts M, Van Broeckhoven C. NanoPack: visualizing and processing long-read sequencing data. Bioinformatics. 2018;34(15):2666–9. 10.1093/bioinformatics/bty149 PMC6061794 29547981

[9] Shabardina V, Kischka T, Manske F, Grundmann N, Frith MC, Suzuki Y, et al. NanoPipe-a web server for nanopore MinION sequencing data analysis. GigaScience. 2019;8(2). 10.1093/gigascience/giy169. 10.1093/gigascience/giy169 PMC6377397 30689855

[10] Bolognini D, Bartalucci N, Mingrino A, Vannucchi AM, Magi A (2019). NanoR: A user-friendly R package to analyze and compare nanopore sequencing data. PloS one.

[11] Ferguson JM, Smith MA. SquiggleKit: A toolkit for manipulating nanopore signal data. Bioinformatics (Oxford, England). 2019;35(24):5372–3. 10.1093/bioinformatics/btz586. 10.1093/bioinformatics/btz586 PMC9887521 31332428

[12] Berndt DJ, Clifford J, editors. Using dynamic time warping to find patterns in time series. Seattle: AAAI Technical Report WS-94-03;1994.

[13] Maaten Lvd, Hinton G (2008). Visualizing data using t-SNE. J Machine Learn Res.

[14] Boccaletto P, Machnicka MA, Purta E, Piatkowski P, Baginski B, Wirecki TK (2018). MODOMICS: a database of RNA modification pathways. 2017 update. Nucleic Acids Res.

[15] Cantara WA, Crain PF, Rozenski J, McCloskey JA, Harris KA, Zhang X (2011). The RNA Modification Database, RNAMDB: 2011 update. Nucleic Acids Res.

[16] Roignant JY, Soller M (2017). m(6)A in mRNA: An Ancient Mechanism for Fine-Tuning Gene Expression. Trends Genet.

[17] Meyer KD, Jaffrey SR (2017). Rethinking m(6)A Readers, Writers, and Erasers. Annu Rev Cell Dev Biol.

[18] Meyer KD, Saletore Y, Zumbo P, Elemento O, Mason CE, Jaffrey SR (2012). Comprehensive analysis of mRNA methylation reveals enrichment in 3’ UTRs and near stop codons. Cell.

[19] Bodi Z, Bottley A, Archer N, May ST, Fray RG (2015). Yeast m6A Methylated mRNAs Are Enriched on Translating Ribosomes during Meiosis, and under Rapamycin Treatment. PloS one.

[20] Liu N, Pan T, Probing (2016). N(6)-methyladenosine (m(6)A) RNA Modification in Total RNA with SCARLET.. Methods Mol Biol.

[21] Motorin Y, Lyko F, Helm M (2010). 5-methylcytosine in RNA: detection, enzymatic formation and biological functions. Nucleic Acids Res.

[22] Frye M, Harada BT, Behm M, He C (2018). RNA modifications modulate gene expression during development. Science.

[23] Wang X, Lu Z, Gomez A, Hon GC, Yue Y, Han D (2014). N6-methyladenosine-dependent regulation of messenger RNA stability. Nature.

[24] Squires JE, Patel HR, Nousch M, Sibbritt T, Humphreys DT, Parker BJ (2012). Widespread occurrence of 5-methylcytosine in human coding and non-coding RNA. Nucleic Acids Res.

[25] Yang X, Yang Y, Sun BF, Chen YS, Xu JW, Lai WY (2017). 5-methylcytosine promotes mRNA export - NSUN2 as the methyltransferase and ALYREF as an m(5)C reader. Cell Res.

[26] Khoddami V, Cairns BR (2013). Identification of direct targets and modified bases of RNA cytosine methyltransferases. Nat Biotechnol.

[27] Sovic I, Sikic M, Wilm A, Fenlon SN, Chen S, Nagarajan N (2016). Fast and sensitive mapping of nanopore sequencing reads with GraphMap. Nat Commun.

[28] Tilgner H, Jahanbani F, Blauwkamp T, Moshrefi A, Jaeger E, Chen F (2015). Comprehensive transcriptome analysis using synthetic long-read sequencing reveals molecular co-association of distant splicing events. Nat Biotechnol.

[29] Cho H, Davis J, Li X, Smith KS, Battle A, Montgomery SB (2014). High-resolution transcriptome analysis with long-read RNA sequencing. PloS one.

[30] Buono P, Plaisant C, Simeone A, Aris A, Shneiderman B, Shmueli G et al, editors. Similarity-based forecasting with simultaneous previews: A river plot interface for time series forecasting. 2007 11th International Conference Information Visualization (IV’07); 2007: IEEE.

[31] Badam SK, Zhao J, Elmqvist N, Ebert DS, editors. Timefork: Mixed-initiative time-series prediction. 2014 IEEE Conference on Visual Analytics Science and Technology (VAST); 2014: IEEE.

