# Supplementary material for: Sequoia: an interactive visual analytics platform for interpretation and feature extraction from nanopore sequencing datasets
Source: BMC Genomics. 2021 Jul 7;22:513. doi: 10.1186/s12864-021-07791-z (PMC8262049; doi:10.1186/s12864-021-07791-z)
Supplement: Supplementary file 1 — Additional file 1: Supplementary Figure 1. Dissection of data units used in the processing of the input FAST5 file. Separated sections showing composition of FAST5 files with Signal list and Events table. Using this Table, 1-mers across each read are annotated with pertaining signals: referred to as Signal instance. All signal instances of 5-mers across reads are condensed into cumulative signals. [file 12864_2021_7791_MOESM1_ESM.pdf]

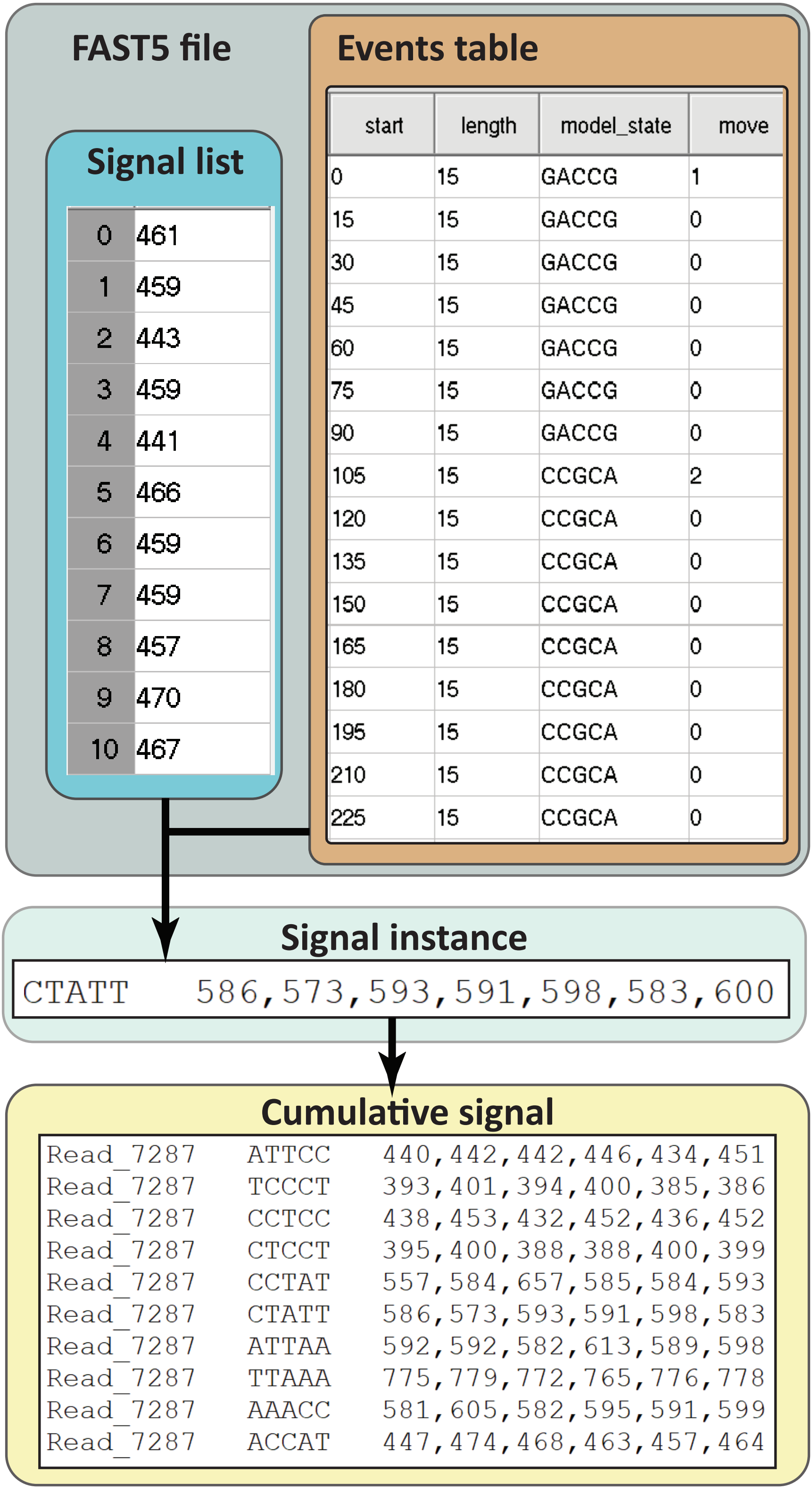
FAST5 file
Events table
start length model_state move
0 15 GACCG 1
15 15 GACCG 0
30 15 GACCG 0
45 15 GACCG 0
60 15 GACCG 0
75 15 GACCG 0
90 15 GACCG 0
105 15 CCGCA 2
120 15 CCGCA 0
135 15 CCGCA 0
150 15 CCGCA 0
165 15 CCGCA 0
180 15 CCGCA 0
195 15 CCGCA 0
210 15 CCGCA 0
225 15 CCGCA 0
Signal list
0 461
1 459
2 443
3 459
4 441
5 466
6 459
7 459
8 457
9 470
10 467
Signal instance
CTATT 586,573,593,591,598,583,600
Cumulative signal
Read_7287 ATTCC 440,442,442,446,434,451
Read_7287 TCCCT 393,401,394,400,385,386
Read_7287 CCTCC 438,453,432,452,436,452
Read_7287 CTCCT 395,400,388,388,400,399
Read_7287 CCTAT 557,584,657,585,584,593
Read_7287 CTATT 586,573,593,591,598,583
Read_7287 ATTAA 592,592,582,613,589,598
Read_7287 TTAAA 775,779,772,765,776,778
Read_7287 AAACC 581,605,582,595,591,599
Read_7287 ACCAT 447,474,468,463,457,464
